# Biocompatible Biphasic Iontronics Enable Neuron-Like Ionic Signal Transmission

**DOI:** 10.34133/research.0294

**Published:** 2024-01-30

**Authors:** Xiaoyi Wang, Aleksandar P. Ivanov, Joshua B. Edel

**Affiliations:** Department of Chemistry, Imperial College London, Molecular Sciences Research Hub, London W12 0BZ, UK.

## Abstract

Biocompatible connections between external artificial devices and living organisms show promise for future neuroprosthetics and therapeutics. The study in *Science* by Zhao and colleagues introduces a cascade-heterogated biphasic gel (HBG) iontronic device, which facilitates electronic-to-multi-ionic signal transduction for abiotic–biotic interfaces. Inspired by neuron signaling, the HBG device demonstrated its biocompatibility by regulating neural activity in biological tissue, paving the way for wearable and implantable devices, including brain–computer interfaces.

Brain–computer interfaces (BCIs) play a crucial role in establishing direct communication between living organs or tissues and external electronic devices [1]. Particularly in neuroprosthetics, these interfaces replace impaired nervous system functions, connecting with the remaining neural network systems. Such BCIs have shown promise in restoring various physiological functions, from sight and hearing to movement, communication, and even cognitive function. More importantly, BCIs have the potential to alleviate and treat conditions such as amyotrophic lateral sclerosis (ALS) and other neurodegenerative diseases [2]. However, ensuring the biocompatibility of external devices with natural neural networks remains a challenge for clinical BCI applications.

In a recent publication in *Science*, Zhao and colleagues introduced a cascade-heterogated biphasic gel (HBG) iontronic material capable of transducing electronic signals into multiple ionic signals, mimicking neuron signaling. This iontronic interface holds the potential to control and modulate the neural activities of implanted biological tissues [3].

Conventional electronic devices rely on electrons or single-type ions as signal carriers, whereas natural neurons use multiple ions or neurotransmitters for processing neural signals [4]. While ions and neurotransmitters were employed as signal carriers in a synthetic nerve [5], the diversity of signal carriers for artificial neurons has never quite matched native ones [6]. Zhao and colleagues addressed this limitation by developing a cascade-heterogated HBG system, which incorporates a microscale ion-enriched internal gel phase (IE phase) and a low-conductivity continuous gel phase (LC phase), as illustrated in Fig. 1.

**Fig. 1.**
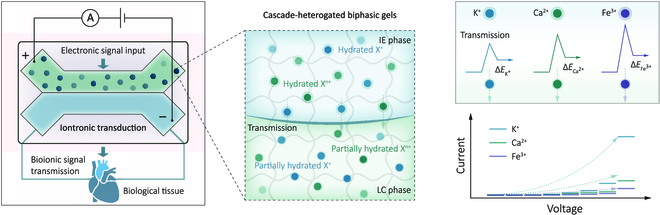
Fabrication of the PAA/Cel hydrogel. (A) Schematic of the developed microstructure from the PAA/Cel hydrogel. (B) Photograph of the PAA/Cel hydrogel. (C) Photographs of the PAA/Cel hydrogel with original and twisting state at −70 °C. (D) A comprehensive comparison between the PAA/Cel hydrogel from this work and previously reported robust counterparts in several key metrics including freezing temperature, compressive strength, tensile strength, toughness, fracture strain, and ionic conductivity.

The IE phase features hydrogel microinclusions designed for storing and transmitting hydrated ions. In contrast, the LC phase functions as an organogel matrix, serving as a partially dehydrated ion transmission medium. When subjected to external electrical field stimulation, this structure initiates ionic cross-medium transmission, prompting the shedding and reconfiguration of cationic hydration shells. These orchestrated processes enhance the distinction among multivariate cations, each experiencing varied cross-interface free energy barriers during ionic transport. This intricate mechanism allows for electronic-to-multi-ionic hierarchical transmission and selective ionic cross-stage transmission.

Zhao and colleagues demonstrated electric field-controlled heterogated ion transport and introduced a chemical-gated structure using crown groups for K^+^ and carboxylic groups for Fe^3+^. This approach would provide an alternative for modulating the transport of ions with the same valence, such as Na^+^ and K^+^, resembling ion channels in cellular membranes [7]. Moreover, the adaptability of HBG materials allows for integration with biorecognition receptors specific to neurotransmitters, including neurotransmitter aptamers [8], thereby expanding the potential for gated transport of organic signal carriers beyond ions.

Biological neuronal signaling relies on the gated transmission of ions and neurotransmitters. The unique capability of HBG systems to convert electronic signals into diverse bioionic signals renders them compatible with biological contexts. This work demonstrated its biocompatibility by successfully regulating cardiomyocyte permeability and modulating cardiac electrical activities in a bullfrog heart. This biocompatible connection holds promise for implanting living organs or tissues with external assistance devices while preserving normal neural activity controls. The iontronic devices developed by Zhao and colleagues could lead to wearable or implantable devices, including advancements in BCIs. Future developments might involve the integration of micro- or nano-fluidics, aiming to enhance transmission performance, reduce device sizes, and improve ion or neurotransmitter selectivity.

## References

[1] Saha S, Mamun KA, Ahmed K, Mostafa R, Naik GR, Darvishi S, Khandoker AH, Baumert M. Progress in brain computer interface: Challenges and opportunities. Front Syst Neurosci. 2021;15:578875. 33716680 10.3389/fnsys.2021.578875 PMC7947348

[2] Vansteensel MJ, Klein E, Thiel G. van, Gaytant M, Simmons Z, Wolpaw JR, Vaughan TM. Towards clinical application of implantable brain–computer interfaces for people with late-stage ALS: Medical and ethical considerations. J Neurol. 2023;270(3):1323–1336. 36450968 10.1007/s00415-022-11464-6 PMC9971103

[3] Chen W, Zhai L, Zhang S, Zhao Z, Hu Y, Xiang Y, Liu H, Xu Z, Jiang L, Wen L. Cascade-heterogated biphasic gel iontronics for electronic-to-multi-ionic signal transmission. Science. 2023;382(6670):559–565. 37917701 10.1126/science.adg0059

[4] Mei T, Zhang H, Xiao K. Bioinspired artificial ion pumps. ACS Nano. 2022;16(9):13323–13338. 36036646 10.1021/acsnano.2c04550

[5] CEG H, Schild VR, Vinals J, Bayley H. Parallel transmission in a synthetic nerve. Nat Chem. 2022;14(6):650–657. 35449216 10.1038/s41557-022-00916-1 PMC7617068

[6] Xiong Y, Han J, Wang Y, Wang ZL, Sun Q. Emerging iontronic sensing: Materials, mechanisms, and applications. Research. 2022;2022: Article 9867378. 36072274 10.34133/2022/9867378 PMC9414182

[7] Acar ET, Buchsbaum SF, Combs C, Fornasiero F, Siwy ZS. Biomimetic potassium-selective nanopores. Sci Adv. 2019;5(2): Article eaav2568. 30783627 10.1126/sciadv.aav2568 PMC6368432

[8] Nakatsuka N, Yang KA, Abendroth JM, Cheung KM, Xu X, Yang H, Zhao C, Zhu B, Rim YS, Yang Y, et al. Aptamer–field-effect transistors overcome Debye length limitations for small-molecule sensing. Science. 2018;362:319–324. 30190311 10.1126/science.aao6750 PMC6663484

